# Running vs. resistance exercise to counteract deconditioning induced by 90-day head-down bedrest

**DOI:** 10.3389/fphys.2022.902983

**Published:** 2022-08-31

**Authors:** Adrien Robin, Linjie Wang, Marc-Antoine Custaud, Jiexin Liu, Min Yuan, Zhili Li, Jean-Christophe Lloret, Shujuan Liu, Xiaoqian Dai, Jianfeng Zhang, Ke Lv, Wenjiong Li, Guillemette Gauquelin-Koch, Huijuan Wang, Kai Li, Xiaotao Li, Lina Qu, Nastassia Navasiolava, Yinghui Li

**Affiliations:** ^1^ Univ Angers, CHU Angers, CRC, INSERM, CNRS, MITOVASC, Equipe CarMe, SFR ICAT, Angers, France; ^2^ State Key Laboratory of Space Medicine Fundamentals and Application, China Astronaut Research and Training Center, Beijing, China; ^3^ Beijing Tiantan Hospital, Medical Capital University, Beijing, China; ^4^ CNES, Toulouse, France; ^5^ CNES, Paris, France

**Keywords:** maximal working capacity, countermeasure, microgravity, HDBR, cardiovascular deconditioning, orthostatic tolerance, V̇O_2_max, oxygen uptake

## Abstract

Spaceflight is associated with enhanced inactivity, resulting in muscular and cardiovascular deconditioning. Although physical exercise is commonly used as a countermeasure, separate applications of running and resistive exercise modalities have never been directly compared during long-term bedrest. This study aimed to compare the effectiveness of two exercise countermeasure programs, running and resistance training, applied separately, for counteracting cardiovascular deconditioning induced by 90-day head-down bedrest (HDBR). Maximal oxygen uptake ( $\dot{\text{V}}$ O_2_max), orthostatic tolerance, continuous ECG and blood pressure (BP), body composition, and leg circumferences were measured in the control group (CON: *n* = 8), running exercise group (RUN: *n* = 7), and resistive exercise group (RES: *n* = 7). After HDBR, the decrease in $\dot{\text{V}}$ O_2_max was prevented by RUN countermeasure and limited by RES countermeasure (−26% in CON *p* < 0.05, −15% in RES *p* < 0.05, and −4% in RUN *ns*). Subjects demonstrated surprisingly modest orthostatic tolerance decrease for different groups, including controls. Lean mass loss was limited by RES and RUN protocols (−10% in CON vs. −5% to 6% in RES and RUN). Both countermeasures prevented the loss in thigh circumference (−7% in CON *p* < 0.05, −2% in RES *ns*, and −0.6% in RUN *ns*) and limited loss in calf circumference (−10% in CON vs. −7% in RES vs. −5% in RUN). Day–night variations in systolic BP were preserved during HDBR. Decrease in $\dot{\text{V}}$ O_2_max positively correlated with decrease in thigh (*r* = 0.54 and *p* = 0.009) and calf (*r* = 0.52 and *p* = 0.012) circumferences. During this 90-day strict HDBR, running exercise successfully preserved $\dot{\text{V}}$ O_2_max, and resistance exercise limited its decline. Both countermeasures limited loss in global lean mass and leg circumferences. The $\dot{\text{V}}$ O_2_max reduction seems to be conditioned more by muscular than by cardiovascular parameters.

## 1 Introduction

Exposure to space environment and its ground-based analogs causes several deleterious effects (Wang et al., 2019; Pavy-Le-Traon et al., 2007). Neuromuscular function is systematically affected, with motor disturbances and decreases in muscle tone, force, and mass (Pavy-Le-Traon et al., 2007; Mounier et al., 2009). This muscle deconditioning may be related to inactivity, gravitational unloading, and metabolic changes (Rynders et al., 2018). The cardiovascular system undergoes deconditioning with a decrease in maximal oxygen uptake ( $\dot{\text{V}}$ O_2_max), tachycardia, and orthostatic intolerance (Capelli et al., 2006; Tank et al., 2011; Fu et al., 2019). Orthostatic intolerance represents a major problem for the return of astronauts to gravity and worsens as the length of a mission increases (Meck et al., 2001), with 20% incidence after 8–16 days of flight and 83% after 129–190 days of flight (Meck et al., 2001; Wang et al., 2019; Vernice et al., 2020). The National Aeronautics and Space Administration (NASA) has included this risk in the Human Research Roadmap on issues to be addressed before future exploration missions (Wood et al., 2019). Another important problem for mission success is the preservation of the overall capability to work, reflected by $\dot{\text{V}}$ O_2_max. Not all astronauts diminish $\dot{\text{V}}$ O_2_max during or following their missions (Moore et al., 2014). But globally in 5- to 6-mo flights, $\dot{\text{V}}$ O_2_max decreases with maximum at first 10–14 days averaging 17%, then rises gradually, remaining however about 15% below pre-flight values (Wang et al., 2019; Moore et al., 2014; Hoffmann et al., 2016).

Maintaining the health and fitness of astronauts is essential, particularly with a view toward deep space missions. Several countermeasures have been tested or used empirically to limit cardiovascular deconditioning and preserve physical performance. Thus, salt and water ingestion prior to landing (Wang et al., 2019; Kozlovskaya et al., 2015) is used to expand plasma volume. A lower body gradient compression garment (Wang et al., 2019; Kozlovskaya et al., 2015) is used to create a cephalad fluid shift during landing and postflight adaptation. Onboard, a lower body negative pressure device (LBNP) (Wang et al., 2019; Kozlovskaya et al., 2015) is used, mainly by Russian crew members, to create a downward fluid shift, simulating the “cardiovascular” effects of orthostasis. A key element in the countermeasures system in long-term flights is physical training and, especially, locomotor training (Grigoriev and Kozlovskaya 2018). Astronauts exercise daily to limit the decrease in cardiorespiratory and muscular fitness (Wang et al., 2019; Kozlovskaya et al., 2015).

Several head-down bedrest (HDBR) investigations have already been conducted to test exercise countermeasures against cardiovascular deconditioning. Resistance exercise alone (flywheel horizontal leg press) did not counteract the decrease in orthostatic tolerance following 90-day HDBR (Belin de Chantemele et al., 2004). Aerobic exercise of moderate intensity and duration seems insufficient to preserve maximal aerobic capacity (Lee et al., 2010). Indeed, mild supine exercise (cycling at 40% $\dot{\text{V}}$ O_2_max for 1 h daily) during 20-day horizontal bedrest did not prevent the loss in $\dot{\text{V}}$ O_2_max in young men (−11% in exercise group vs. −14% in controls) (Suzuki et al., 1994). However, extensive aerobic exercise training combined with LBNP is able to protect upright $\dot{\text{V}}$ O_2_peak, as shown with a 6-day/wk 40-min interval treadmill within the LBNP during 30-day HDBR in men (Lee et al., 2007) and women (Lee et al., 2009), compared to their nonexercising twins.

Separate applications of running and resistive exercise modalities have never been directly compared during long-term HDBR. It is important to understand the separate effects of locomotor and resistive exercise to optimize countermeasure prescriptions, to individually adapt the proportion of modalities for different moments of missions and different aspects of deconditioning, and, in case of difficulties, to use a combination of countermeasures because of crew time, spacecraft volume, technology, equipment issues, etc.

### 1.1 Objective

We aimed to compare the effectiveness of two exercise countermeasure programs, running and resistance, applied separately, for counteracting cardiovascular deconditioning in healthy young men during a 90-day HDBR.

## 2 Materials and methods

### 2.1 Subjects

A total of 22 healthy Asian nonathletic men (with maximal oxygen uptake, $\dot{\text{V}}$ O_2_max, not exceeding 55 ml kg^−1^. min^−1^) were studied before, during, and after 90 days of 6° HDBR. The volunteers were stratified by baseline $\dot{\text{V}}$ O_2_max and then randomized into 3 groups in a balanced manner to minimize group differences in $\dot{\text{V}}$ O_2_max: control without countermeasures (CON, *n* = 8); running countermeasure (vertical treadmill running, RUN, *n* = 7); and resistance exercise countermeasure (RES, *n* = 7). The baseline anthropometric data are summarized in Table 1. The groups were not significantly different in age, height, or weight.

**TABLE 1:** Baseline anthropometric data.

	CON (*n* = 8)	RUN (*n* = 7)	RES (*n* = 7)	*p* value
Age, yr	30 ± 2	34 ± 2	32 ± 2	0.46
Height, cm	170 ± 1	170 ± 1	168 ± 2	0.82
Weight, kg	64 ± 3	65 ± 2	59 ± 3	0.24
V̇O_2_max, ml.kg^−1^.min^−1^	32.6 ± 2.2	33.0 ± 1.6	31.3 ± 2.3	0.84
V̇O_2_max, L.min^−1^	2.1 ± 0.1	2.1 ± 0.1	1.9 ± 0.1	0.41
Lean mass, kg	55.1 ± 2.7	55.2 ± 1.9	50.1 ± 1.8	0.24
Fat mass, kg	9.0 ± 0.5	9.6 ± 1.6	9.2 ± 1.7	0.96

Data are mean ± SEM; One-way ANOVA, did not reveal significant differences between groups.

The protocol was approved by the ethics committee of the Astronaut Center of China (ACC). All the procedures and risks associated with this experiment were explained to the subjects, and written consent was obtained from each participant.

### 2.2 Study design

**FIGURE 1:**
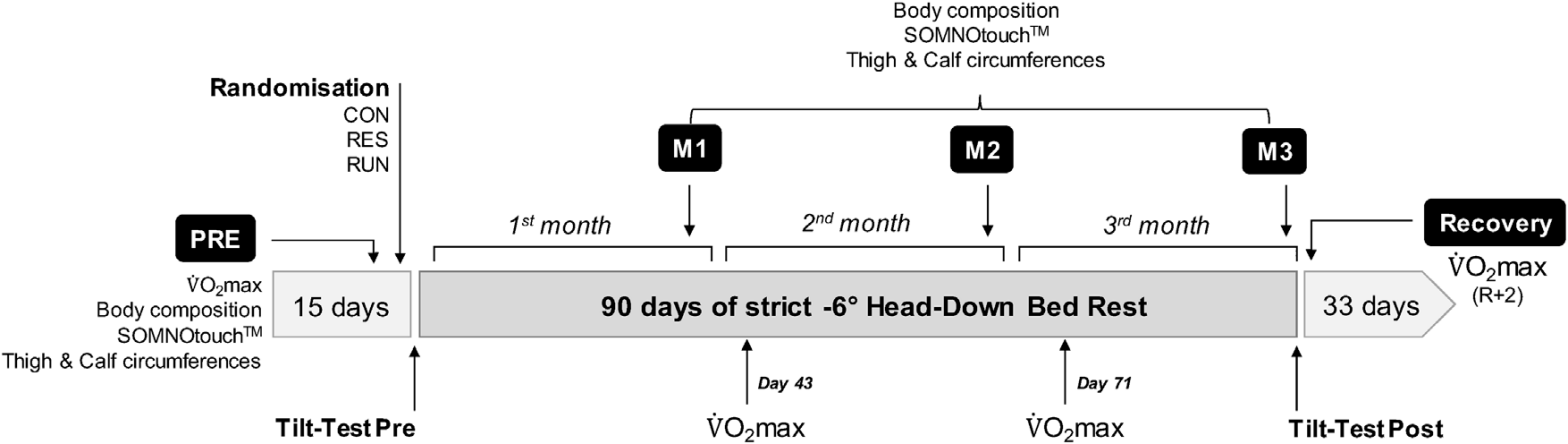
Study chronology with performed measurements. The experiment consisted of 15 days of baseline measurements (Pre), followed by 90 days of strict head-down bedrest where the subjects remained in a 6° head-down posture full time and 33 days of recovery (R + *x*).

The study was conducted at the Space Institute of Southern China (SISC, Shenzhen, China) in 2019. Baseline measurements (Pre) were started 15 days before bedrest (ambulatory subjects freely moving in the facility), and recovery measurements (R) ended 33 days after bedrest (Figure 1). Strict bedrest lasted 90 days. Coffee, tea, and nicotine were prohibited during the experiment. The lights-off period was set from 22:30 to 06:30. All routine activities (toilet, meals, etc.) and height and weight measurements were performed in a 6° head-down posture. Subjects were allowed to change position but were required to keep at least one shoulder in contact with the bed. Muscle activity of the legs was not allowed outside of the specific countermeasures and protocols. Subjects were observed continuously by video to ensure compliance. For operational reasons, daily caloric intake was fixed independently of body mass at 2,700 kcal for CON and 2,800 kcal for RUN and RES, with 50%–65% covered by carbohydrates, 20%–35% covered by fat, and 12%–15% covered by protein (with 60% of animal source and 40% of vegetable source). Water intake was *ad libitum*. Sodium intake was 1.5–3 g/day.

### 2.3 Exercise countermeasure protocol

#### 2.3.1 Running exercise countermeasure

**FIGURE 2:**
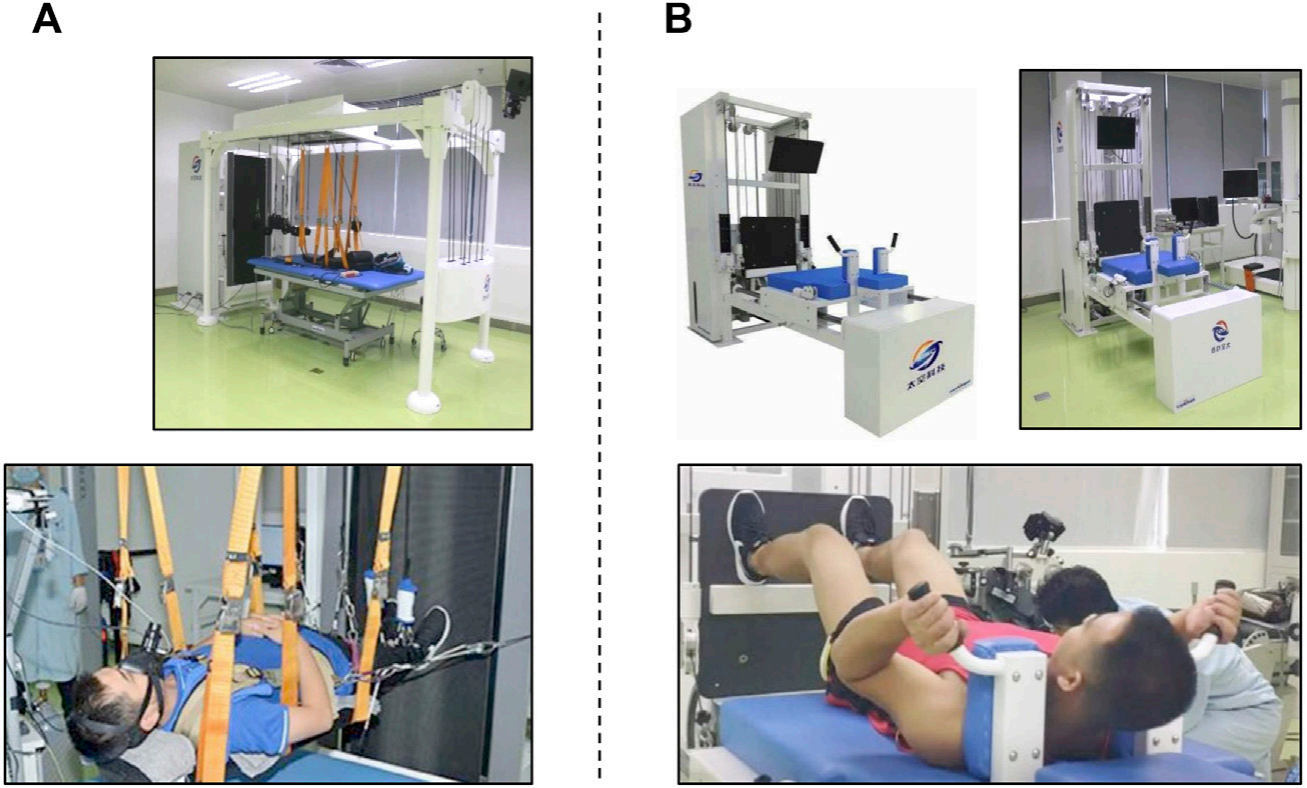
Exercise countermeasures. Treadmill training **(A).** A vertically tilted treadmill allows the subject to perform moderate- and high-intensity running while remaining supine. Exercise protocol: moderate running (60%–80% $\dot{\text{V}}$ O_2_max) for 30 min 3x/week and 4 sets of high-intensity running (90% $\dot{\text{V}}$ O_2_max) for 4 min once a week. Resistance training **(B)**. A vertically tilted resistance exercise platform allows the subject to perform resistance exercises (squats, heel raises, shoulder shrugs, and curls) while remaining supine. Exercise protocol: 3 sets of 10–12 repetitions (60%–80% max voluntary contraction), 45 min 3x/week.

The RUN group used a vertical treadmill, which allowed exercise to be performed while lying down horizontally (Figure 2A). A shoulder-and-waist harness was used to load the exercising subjects. The subjects ran continuously at about 70%–80% of their $\dot{\text{V}}$ O_2_max (80%–89% of their HR max) for 30 min three times a week (Monday, Wednesday, and Saturday). In addition, once a week (Friday), they performed a high-intensity run over 4 sets of 4 min (90% $\dot{\text{V}}$ O_2_max) followed by 3 min of active rest (40% $\dot{\text{V}}$ O_2_max). In the first 2 weeks, subjects were running at 60%–80% $\dot{\text{V}}$ O_2_max with a load of 60%–80% body mass for adaptation. The external load was maintained at about 80% body mass after adaptation.

Continuous running and high-intensity run sets were performed in the motor-powered treadmill mode. Heart rate and running speed were controlled. Exercise levels were adjusted to the desired percentage of maximal HR by modifying running speed at a scale of 0.5 km/h.

#### 2.3.2 Resistance exercise countermeasure

The RES group used a flywheel exercise device, which was mounted on a supine resistance exercise platform to allow exercising while lying down (Figure 2B). This supine resistance exercise platform was developed to simulate the exercise mode in orbit without the action of body weight. We wanted this platform to provide free weight or flywheel resistance. This platform has two kinds of resistance force. One comes from the constant resistance block. Another resistance force comes from the flywheel. Its force is transmitted by a tension band. The output resistance splits by a fixed pulley and then each end connects to a moving pulley. Through this pulley assembly, we can have the bilateral resistance output. In fact, using a flywheel can complete exercise in the supine posture. As we have established the platform and the posture can be easily controlled in this platform, we have chosen to use the platform to complete this experiment.

Subjects were positioned on a moveable platform with the shoulder pads and hand grips. The subjects exercised for 45 min three times a week (Monday, Wednesday, and Friday). They performed squats, heel raises, shoulder shrugs, and curls at 60%–80% of maximal voluntary contraction (MVC) in 3 sets of 10–12 repetitions. For squats, the knee flexion angle was from ∼90° to ∼170°, and repetition of concentric to eccentric contraction was about 2–3 s. For heel raises, the subject placed his forefoot on the platform, did ankle plantar flexion, and maintained it for about 2 s, letting the heel drop when he felt obvious stretching of the gastrocnemius. For shrugs, the subject lifted the shoulders as high as he could and held contraction for 1 s and then slowly returned to the starting position. For curls, the subject lifted up the holding bar in about 2 s and then returned to the starting position in about 2 s. Subjects were given visual feedback, ensuring that the subjects were performing the exercise in the desired range of motion and speed. As the load of the flywheel is tightly related to the speed of action performance, the exercise intensity could not be finely controlled. The primary training load was set at 60%–80% of pre-HDBR MVC, and then the maximal loads were rising across daily training. When the maximal load was greater than 80% MVC, the loads were controlled at the range of 75%–125% of the actual maximal loads.

During bedrest, the loads were adjusted monthly according to MVC assessment results. MVC was determined for squats, as mean maximal contraction for three repetitions. It was planned to adjust resistance exercise intensity if MVC dropped below 75% of the pre-HDBR baseline, but this did not happen.

### 2.4 Maximal oxygen uptake test

An incremental dynamic leg exercise test on a cycle ergometer (Shanxi Orient Health Industry Co., Ltd., China) was performed in the -6° position at Pre, D43, D71, and R + 2 to determine maximal oxygen uptake ( $\dot{\text{V}}$ O_2_max). Breath-by-breath $\dot{\text{V}}$ O_2_ was recorded using a metabolic cart (JAEGER^®^ MasterScreen CPX system, Germany).

The subjects rested for 5 min and then cycled for 2 min at 50 W, followed by an increase of 50 W every 2 min until 150 W, which was then changed to an increase of 25 W every 2 min until the subject reached the maximal heart rate (HR) (220—age) or maximal power of 300 W or 230 mmHg of systolic blood pressure (SBP) or peak exertion or wanted to stop.

### 2.5 Tilt test

A tilt test was performed before and immediately after the end of bedrest (first return to upright), in the morning or in the afternoon (the same sequence of subjects was used at Pre and Post), at least 1 h after the meal, using an automatic tilt table (Beijing Juchi Pharmaceutical Technology Co., Ltd., China). The subject remained in a horizontal position for 10 min, and then supine data were acquired for 10 min. The subject was then tilted up to 75° for a maximum of 30 min. Criteria for stopping the test earlier were symptoms such as excessive sweating, pallor, vertigo, nausea, or a sudden drop in BP (SBP drop > 25 mmHg/min, or DBP drop > 15 mmHg/min, or SBP < 70 mmHg) or HR drop > 15 beats/min. Subjects completing the full 30 min of tilt were considered finishers.

During the test, orthostatic tolerance time (OTT) was measured. Subjects were monitored in real time using a task-force monitor (CN-system). Hemodynamic indices—blood pressure (SBP and DBP), HR, stroke volume (SV; assessed by impedance cardiography), and total peripheral resistance (TPR)—were noted every 5 min; the ECG was recorded.

Autonomic cardiac modulation was assessed from the ECG recording *via* spectral heart rate variability (HRV) analysis markers—normalized low-frequency (LF) and high-frequency (HF) spectrum power and the LF-to-HF ratio [for details see Coupé et al. (2011)]. The last 5 min of stable supine and tilt recordings (before the onset of intolerance if it occurred) were selected for this analysis.

### 2.6 Continuous monitoring of electrocardiogram, blood pressure, and actimetry

Measurements were performed using the SOMNOtouch™ (SOMNOmedics^®^, Randersacker, Germany) at four time points: once at Pre and once at the end of each month of HDBR (M1, M2, and M3). This system allows a beat-by-beat estimation of blood pressure based on the measurement of the pulse transit time (Bilo et al., 2015).

The SOMNOtouch™ was placed a few hours (0–2 h) before bedtime and was removed the following evening. Brachial blood pressure for calibration was measured using an Omron automatic blood pressure monitor. Cardiovascular variables (R-R interval, SBP, and DBP) and activity (wrist monitor) were recorded continuously for 21 h, from 21:00 to 18:00. The average values per hour were extracted.

To calculate “day” and “night” mean values, the 23:00–05:00 period was considered “night,” and 07:00–17:00 was considered “day.” The 22:00 and 06:00 points were excluded from the averages to avoid artifacts during these transition periods.

### 2.7 Body mass, body composition, and fluid compartments

Bioimpedance measurements using a multifrequency impedance device (ImpediMed SFB7, Carlsbad) were performed at Pre, D30, D57, and D85 in the morning before breakfast and after voiding. The subjects were weighed prior to measurements, and body mass data were used for calculations. Total body water (TBW), extracellular fluid (ECF), intracellular fluid (ICF), fat mass (FM), and lean mass (LM) were estimated.

### 2.8 Thigh and calf circumferences

Thigh and calf circumferences were measured in the −6° position using inelastic tapeline with the same topography, respectively, to the distal apex of the patella at Pre, D28, D56, and D84. Measurements for the two legs were averaged.

### 2.9 Statistical analysis

Data are presented as the mean ± SEM. Three-way ANOVA or 2-way ANOVA for repeated measures was used, with bedrest (measurement time points) and position (supine and tilt) as the within-subject factors and the countermeasure (CON, RES, and RUN) as the between-subject factor. Statistically significant differences were further analyzed by two-sided pairwise multiple comparisons with Sidak correction. Relationships between variables were examined using the Pearson correlation coefficient (*r*). Orthostatic tolerance (finisher and nonfinisher numbers across groups) was analyzed using the chi-square test. Differences were considered statistically significant when the adjusted *p* ≤ 0.05. Analyses were performed using Prism GraphPad 9.0.2.

## 3 Results

### 3.1 General data

All subjects completed the entire protocol without significant medical or psychological issues. All subjects completed all exercise sessions. HR and BP remained within normal limits.

### 3.2 Maximal oxygen uptake

With HDBR, $\dot{\text{V}}$ O_2_max declined by 26% in CON (from 32.6 ± 2.2 ml kg^−1^. min^−1^ at Pre to 24.2 ± 1.9 at R + 2). Of note, most of this decrease (−21%) was already achieved at D43.

**FIGURE 3:**
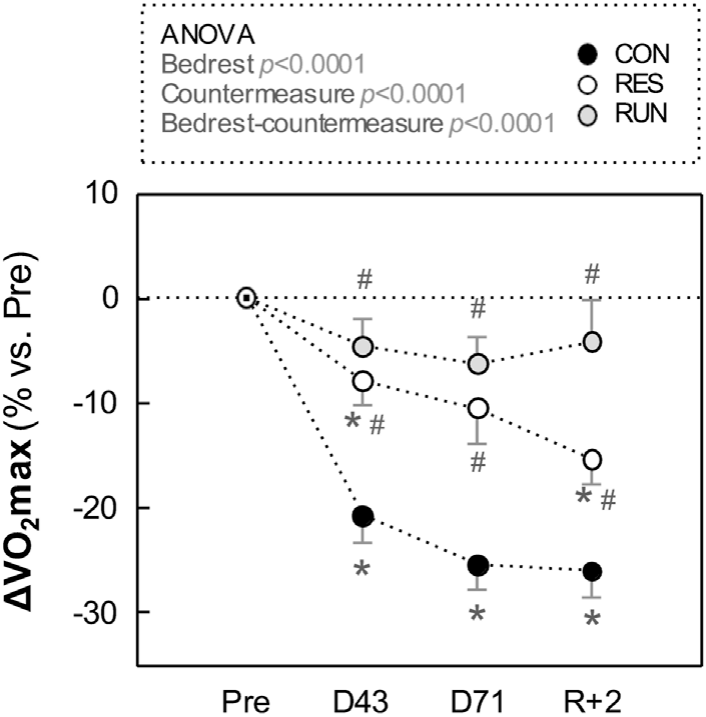
Evolution in maximal oxygen uptake (V̇O_2_max) during HDBR. Measurements were performed in the –6° position using a bicycle ergometer test before (Pre), during (at day 43 and day 71), and after (R + 2: 2nd day of recovery) HDBR. The sample size was *n* = 8/CON, *n* = 7/RES, *n* = 7/RUN. Data are presented as the mean ± SEM. *p < 0.05 vs. Pre; #*p* < 0.05 vs. CON.

In RES, $\dot{\text{V}}$ O_2_max declined 15% post-HDBR (from 31.3 ± 2.3 ml kg^−1^. min^−1^ at Pre to 26.2 ± 1.4 at R + 2). In RUN, $\dot{\text{V}}$ O_2_max was preserved (nonsignificant 4% decrease, from 33.0 ± 1.6 ml kg^−1^. min^−1^ at Pre to 31.4 ± 1.4 at R + 2) (Figure 3; Supplementary Data S1).

### 3.3 Tilt test

#### 3.3.1 Orthostatic tolerance

**FIGURE 4:**
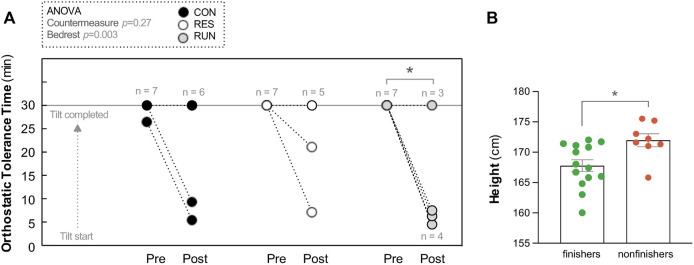
Orthostatic tolerance during a 30-min 75° head-up tilt test. Orthostatic tolerance time (OTT) **(A)**. Tilt was performed before (Pre) and immediately after (Post) 90-day head-down bedrest. Each circle represents the individual OTT. *p < 0.05 vs. OTT Pre. Body height of finishers vs. non-finishers post-HDBR **(B)** (all groups pooled together). Data are presented as the mean ± SEM, *p < 0.05 vs. finishers.

Pre-HDBR, all subjects finished a 30-min upright period, except one in the CON group who became intolerant at the 27th min. Post-HDBR, there were 2 non-finishers out of 8 in CON, 2 out of 7 in RES, and 4 out of 7 in RUN (Figure 4A). The number of nonfinishers post-HDBR did not differ statistically significantly across the groups (chi-square test *p* = 0.38). The post-HDBR orthostatic tolerance time statistically significantly decreased only for RUN and was 24 ± 4 min for CON, 25 ± 3 min for RES, and 16 ± 5 min for RUN, without statistically significant difference between groups. Irrespective of the group, post-HDBR nonfinishers (*n* = 8, height 172 ± 1 cm) were significantly taller than finishers (*n* = 14, height 168 ± 1 cm) (unpaired *t*-test *p* = 0.01; Figure 4B).

#### 3.3.2 Hemodynamic and autonomic responses to tilt

**FIGURE 5:**
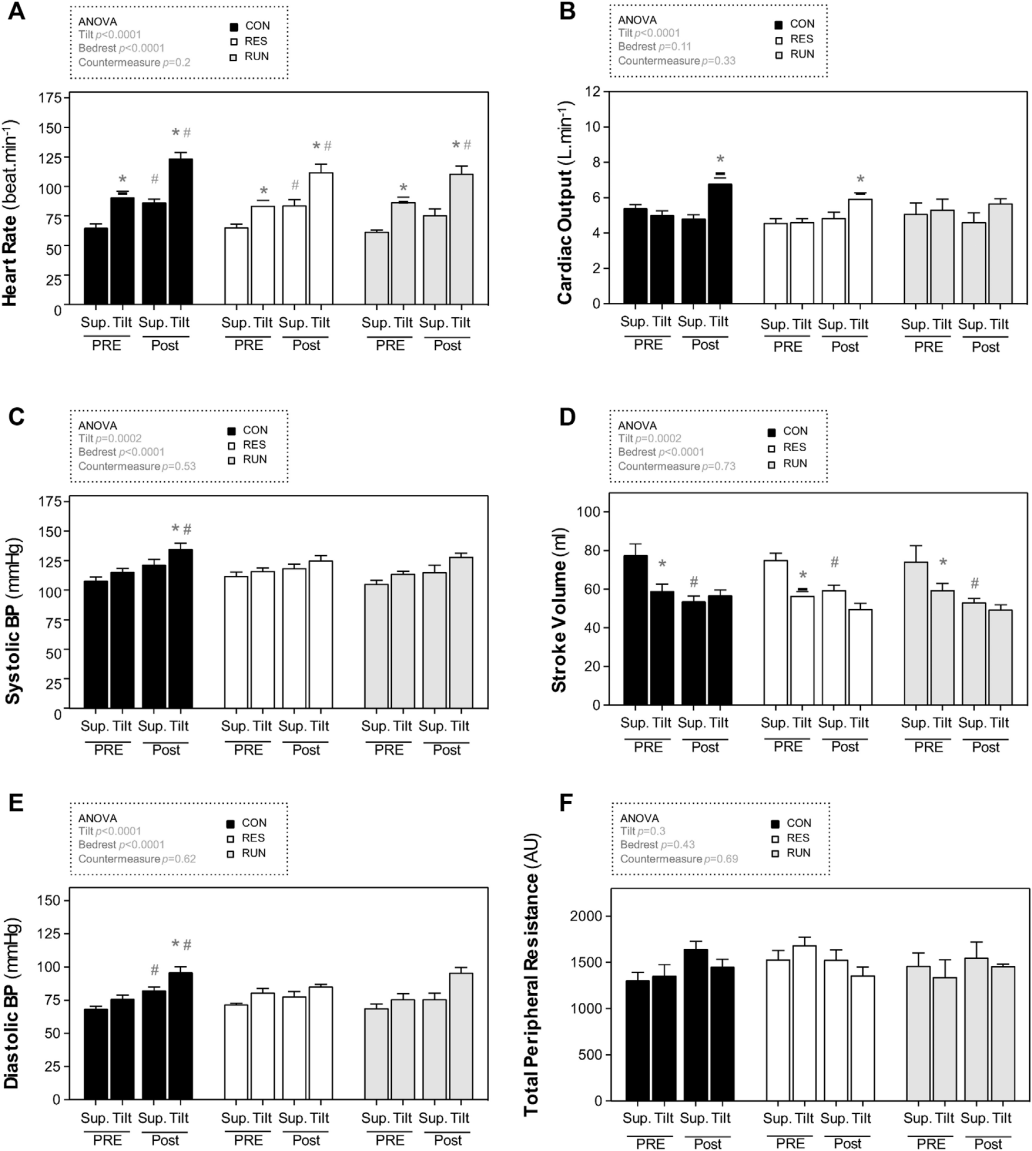
Hemodynamic responses to a 30-min 75° head-up tilt test. The tilt test was performed before (Pre) and immediately after (Post) 90-day head-down bedrest. End-supine (Sup) and end-tilt (Tilt) heart rate **(A)**, cardiac output **(B)**, systolic blood pressure **(C)**, stroke volume **(D)**, diastolic blood pressure **(E)**, and total peripheral resistance **(F)** are shown. The sample size was *n* = 8/CON, *n* = 7/RES, *n* = 7/RUN. Data are presented as the mean ± SEM. *p < 0.05 vs. Supine; ^#^ *p* < 0.05 vs. Pre.

Before bedrest, upright positioning induced the expected changes in central hemodynamics (increased HR and decreased SV), as well as in HRV spectral power (decreased HF, increased LF, and increased LF/HF ratio). SBP and DBP showed a slight increase of 7–8 mmHg, without statistical significance (Figures 5C,E; Table 2).

**TABLE 2:** HRV analysis results during the tilt test.

	Group	Pre-HDBR		Post-HDBR		Global ANOVA
		Supine	Tilt	Supine	Tilt	
LF *normalized*	CON	0.46 ± 0.06	0.80 ± 0.06^*#*^	0.73 ± 0.06*	0.78 ± 0.07	*Tilt p < 0.0001*
	RES	0.34 ± 0.05	0.83 ± 0.03^*#*^	0.56 ± 0.07*	0.86 ± 0.05^*#*^	*Bedrest p = 0.002*
	RUN	0.50 ± 0.06	0.82 ± 0.07^*#*^	0.69 ± 0.06	0.85 ± 0.03^*#*^	*CM p = 0.45*
HF *normalized*	CON	0.54 ± 0.06	0.20 ± 0.06^*#*^	0.27 ± 0.06*	0.22 ± 0.07	*Tilt p < 0.0001*
	RES	0.66 ± 0.05	0.17 ± 0.03^*#*^	0.44 ± 0.07*	0.14 ± 0.05^*#*^	*Bedrest p = 0.002*
	RUN	0.50 ± 0.06	0.18 ± 0.07^*#*^	0.31 ± 0.06	0.15 ± 0.03^*#*^	*CM p = 0.45*
LF/HF ratio	CON	1.00 ± 0.21	7.48 ± 2.23^*#*^	3.97 ± 0.92*	6.99 ± 1.99	*Tilt p < 0.0001*
	RES	0.59 ± 0.14	6.58 ± 1.78	1.72 ± 0.55	11.28 ± 3.61^*#*^	*Bedrest p = 0.17*
	RUN	1.17 ± 0.25	9.81 ± 2.84^*#*^	2.94 ± 0.68	7.60 ± 1.79	*CM p = 0.94*

Data are mean ± SEM; *p < 0.05 vs. Pre; ^#^ *p* < 0.05 vs. Supine; LF, low-frequency band; HF, high-frequency band; CM, countermeasure.

Postbedrest (R0), supine measurements showed a ∼34% increase in HR (from 65 ± 3 to 87 ± 3 bpm in controls), a ∼31% decrease in SV (from 78 ± 6 ml to 54 ± 3 ml in controls), and a 3-fold increase in the LF/HF ratio. Supine BP slightly increased (by 8–15 mmHg), significantly for DBP; supine TPR and CO were not significantly modified. Countermeasures had no significant effect (Figure 5; Table 2).

Postbedrest upright measurements showed a ∼36% increase in upright HR compared to Pre (from 91 ± 5 to 124 ± 5 bpm in controls). Upright BP increased (SBP from 116 ± 3 to 135 ± 5 mmHg and DBP from 76 ± 3 to 96 ± 4 mmHg in controls). Unlike that at prebedrest, postbedrest upright SV (already low when supine) did not further decrease with orthostasis (from 59 ± 3 to 57 ± 3 ml in controls). However, we observed an increase in post-BR upright CO. The LF/HF ratio post bedrest was already increased when supine, and its further increase in response to orthostasis was blunted post-HDBR. Countermeasures had no significant effect on hemodynamic and autonomic responses to tilt (Figure 5; Table 2).

### 3.4 Continuous monitoring of electrocardiogram, blood pressure, and actimetry

**FIGURE 6:**
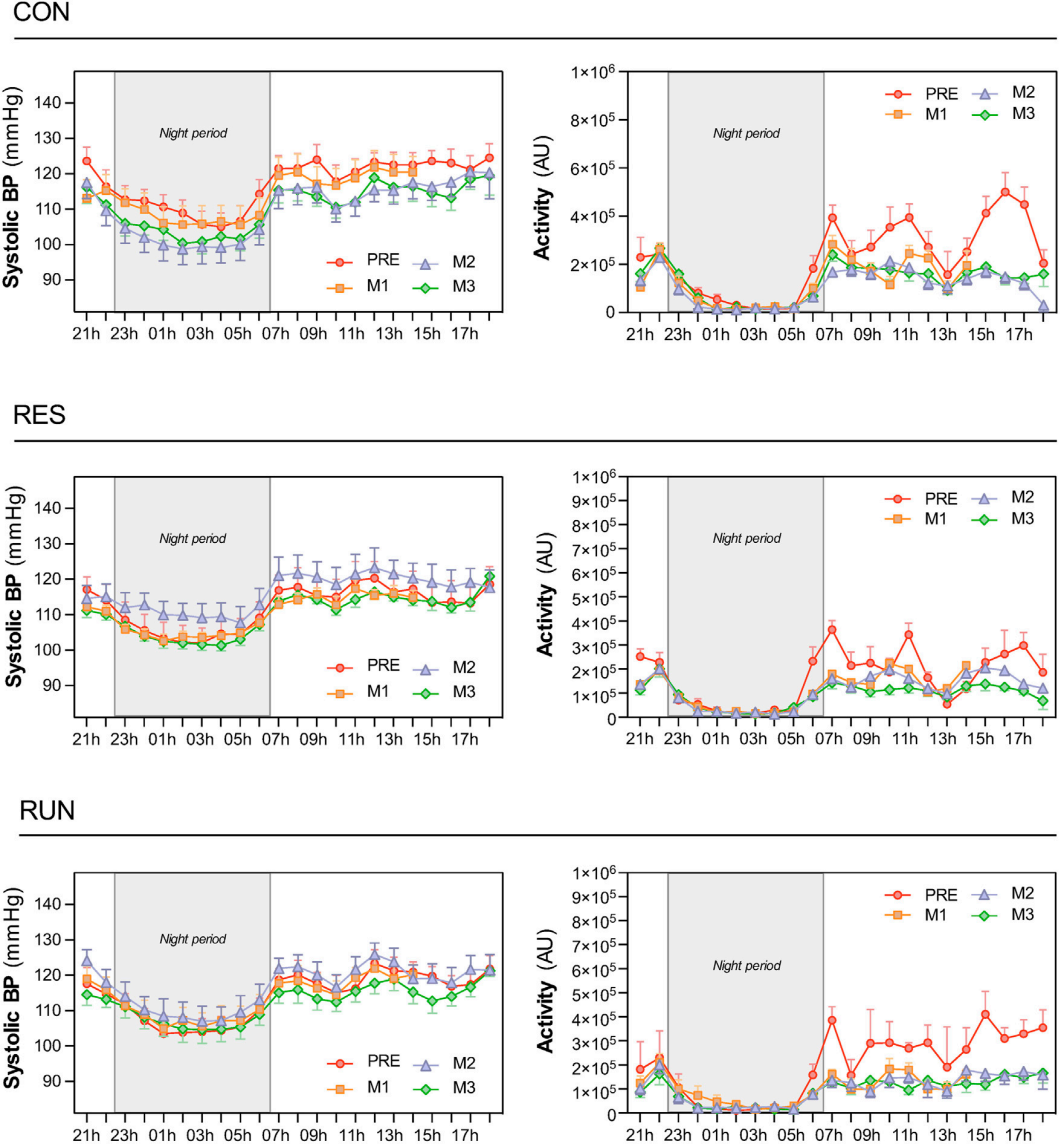
Circadian patterns of systolic blood pressure and physical activity. Recordings were performed using the SOMNOtouch™ device over 21 h, before HDBR and at the end of each month (M1, M2, and M3). The night period was 22:30–06:30. The sample size was *n* = 8/CON, *n* = 7/RES, *n* = 7/RUN. Data are presented as the mean ± SEM.

**FIGURE 7:**
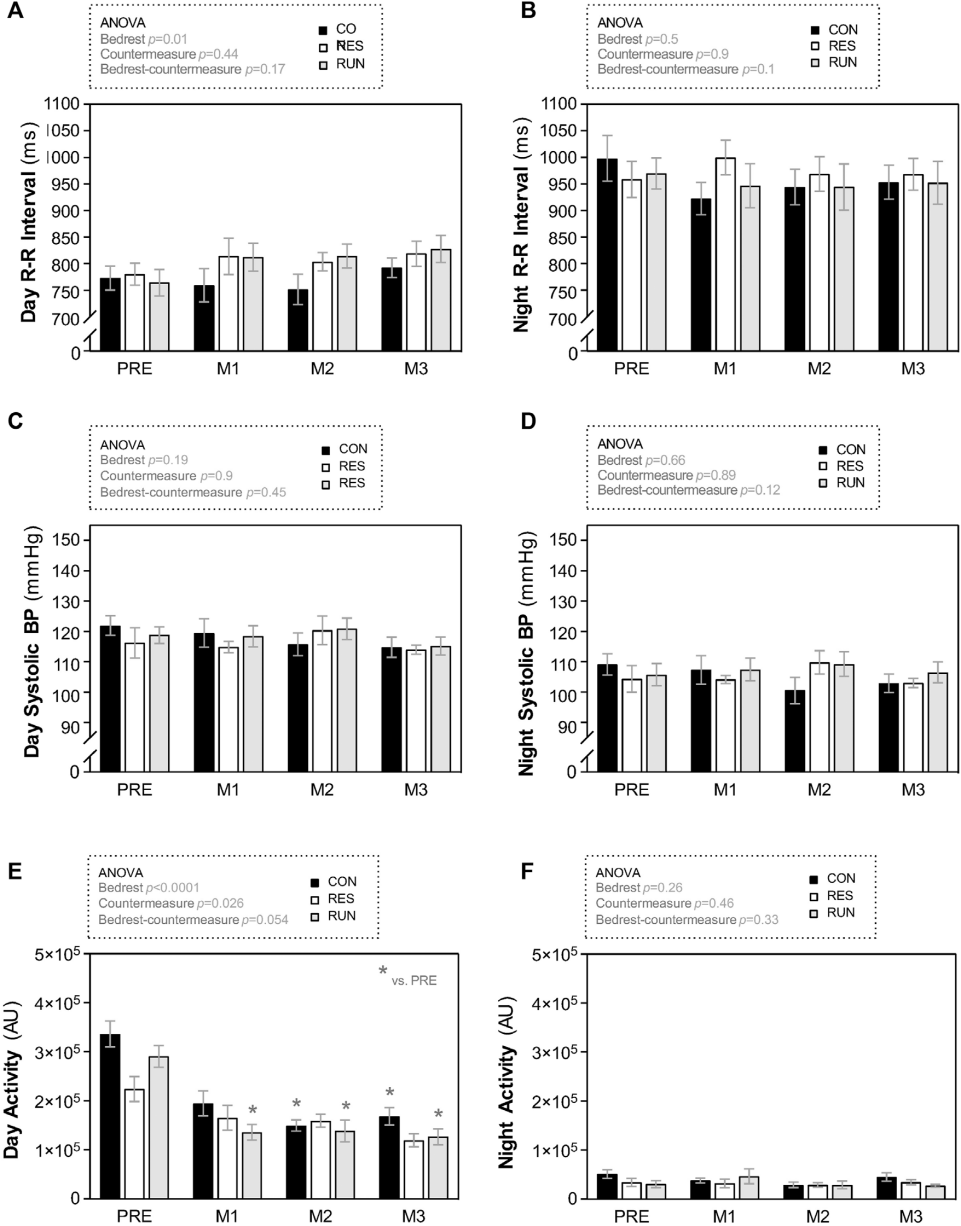
Evolution in fluid compartments, body mass, and body composition during HDBR. Fluid compartments **(A,C,D)** body mass used for bioimpedance measurements **(B)**, and body composition **(E,F)** were estimated by bioimpedance measurements using a multifrequency impedance device. The subjects were weighed prior to measurement, and body mass data used for calculations. The sample size was *n* = 8/CON, *n* = 7/RES, *n* = 7/RUN. Data are presented as the mean ± SEM. *p < 0.05 vs. Pre; ^#^ *p* < 0.05 vs. CON.

Circadian profiles of BP (shown for SBP, Figure 6) and HR (not shown) demonstrated the expected circadian variance, seemingly not much affected by HDBR. For SBP, day–night difference was preserved (nocturnal SBP dipping of 10 ± 1% or ∼12 mmHg throughout the protocol). For the R-R interval, the day–night difference was somewhat flattened under HDBR (from 225 ± 23 ms at Pre to 161 ± 17 ms at M3 in CON, corresponding to decrease in nocturnal HR dipping from 22 ± 1% to 17 ± 1%) (Figures 6, 7A,B).

Circadian BP decreased during HDBR in CON (reduction of 6–7 mmHg for systolic), although without reaching statistical significance for “day” and “night” mean values. In the countermeasure groups, this decreasing pattern was less evident, although the SBP curve still seemed to be lowest at M3.

The activity level dropped as expected during bedrest and was extremely reduced at night (Figures 6, 7E,F).

The R-R interval and SBP, as estimated by “day” and “night” mean values, did not differ between groups during bedrest (Figures 7A–D).

### 3.5 Body mass, body composition, and fluid compartments

**FIGURE 8:**
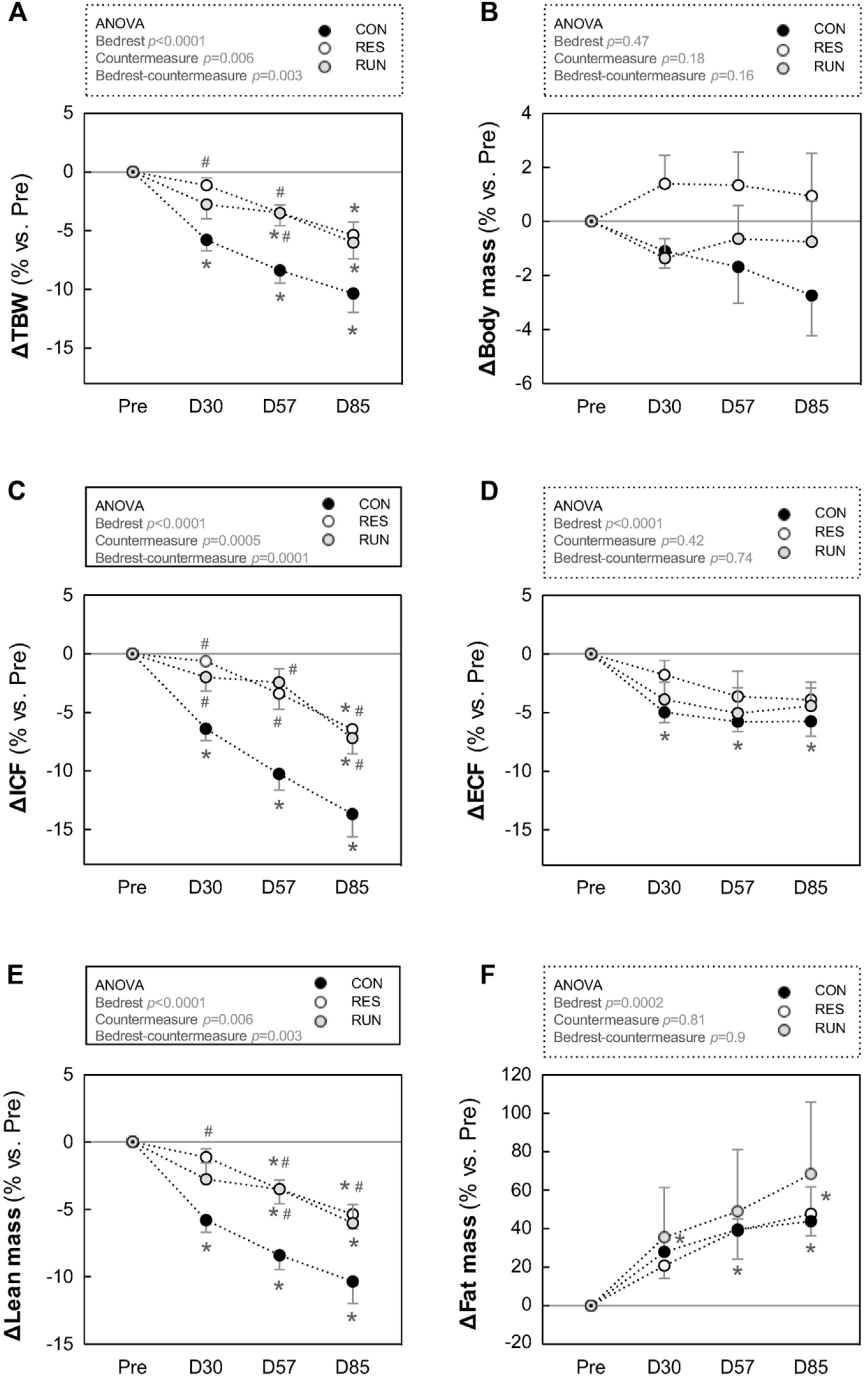
Average day and night R-R interval **(A,B)**, systolic blood pressure **(C,D)**, and physical activity **(E,F)**. To calculate mean “day” and “night” values, 23:00–05:00 was considered “night,” and 07:00–17:00 was considered “day.” The 22:00 and 06:00 points were excluded from the averages to avoid artifacts during these transition periods. Recordings were performed before HDBR and at the end of each month (M1, M2, and M3). The sample size was *n* = 8/CON. Data are presented as the mean ± SEM. *p < 0.05 vs. Pre.

Results are presented as percentage changes in Figure 8 and as changes in native scales in Supplementary Data S2. The body mass of the CON tended to progressively decrease throughout the HDBR (∼2 kg loss at D85), although without reaching statistical significance. Body mass in the countermeasure groups remained rather stable (Figure 8B).

LM in CON gradually decreased during HDBR. LM loss was 2–4 kg in the first month and 5–7 kg at the end of HDBR. This loss in LM was reduced by half in both countermeasure groups (10% loss in CON vs. 5%–6% loss in RES and RUN at the end of HDBR) (Figure 8E).

FM gradually increased during HDBR without a significant difference between groups. By the end of HDBR, subjects gained 3–4 kg in FM (∼9 kg of estimated FM at baseline vs. ∼12–13 kg at D85) (Figure 8F).

TBW in CON gradually decreased, by ∼6% at M1 and ∼10% at M3. This decrease occurred initially (at M1) at the expense of both ECF and ICF compartments and then (at M2–M3) mostly at the expense of the ICF compartment (Figure 8A).

Both countermeasures reduced the losses in TBW and ICF by half (TBW: 10% loss in CON vs. 5%–6% loss in RES and RUN at the end of HDBR; ICF: 14% loss in CON vs. 6%–7% loss in RES and RUN at the end of HDBR). The loss in ECF (4–6% at M3) was not significantly different between groups (Figures 8C,D).

### 3.6 Thigh and calf circumferences

**FIGURE 9:**
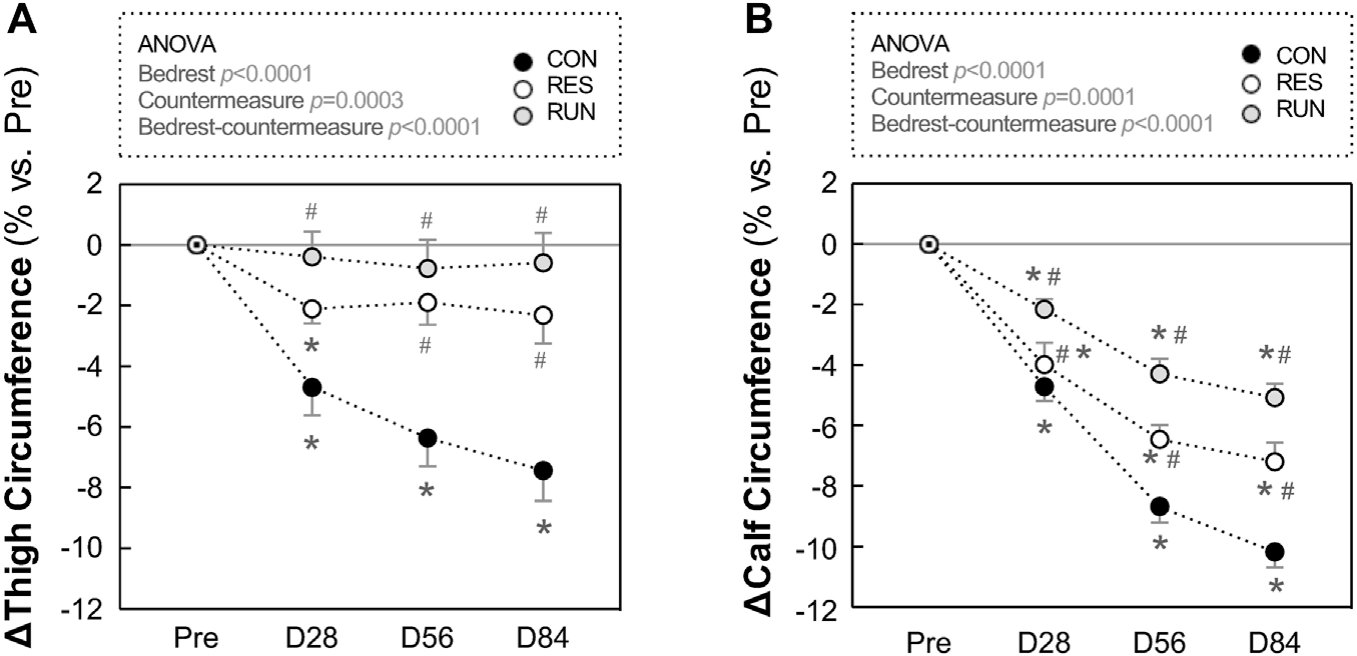
Evolution in thigh and calf circumferences during HDBR. Thigh **(A)** and calf **(B)** circumferences were measured in the –6° position. The sample size was *n* = 8/CON, *n* = 7/RES, *n* = 7/RUN. Data are presented as the mean ± SEM. *p < 0.05 vs. Pre; ^#^ *p* < 0.05 vs. CON.

Results are presented as percentage changes in Figure 9 and as changes in native scales in Supplementary Data S3. Thigh and calf circumferences progressively decreased during HDBR. Most of the changes occurred in the first 2 months and reached 7.4 ± 1.0% loss for the thigh and 10.2 ± 0.5% loss for the calf in the CON group at the end of bedrest. Exercise countermeasures counteracted this decrease, more efficiently at the thigh than at the calf (loss in thigh circumference at M3 was nonsignificant for both countermeasure groups), and more efficiently in RUN than in RES (average loss in calf circumference at M3 was −10.2% in CON, −7.2% in RES, and −5.1% in RUN) (Figure 9).

### 3.7 Correlations between changes in maximal oxygen uptake and muscular and cardiovascular variables

To gain insight into parameters that could be related to the pre-to-post changes in $\dot{\text{V}}$ O_2_max, we performed correlation analysis. Data were expressed as percentages of Pre values. Pairs of data for all 22 subjects from three groups were analyzed together.

**FIGURE 10:**
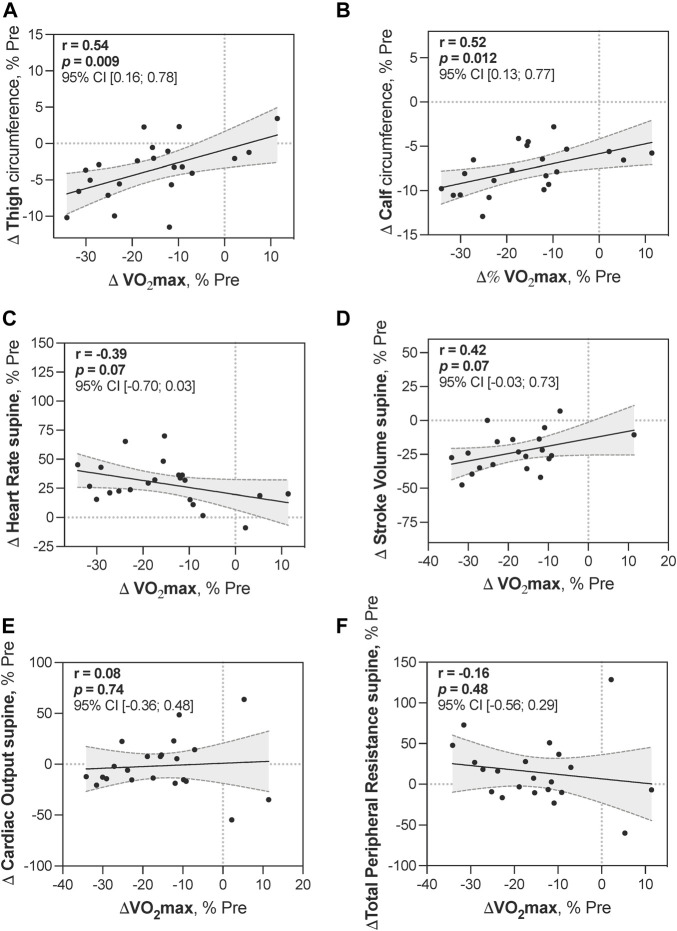
Correlations between changes in V̇O_2_max and muscular and cardiovascular variables. Correlations between relative changes in $\dot{\text{V}}$ O_2_max at R + 2 and relative decreases in thigh **(A)** and calf **(B)** circumferences at D84; relative changes in supine heart rate **(C)**, stroke volume **(D)**, cardiac output **(E)**, and total peripheral resistance **(F)** measured during the tilt test immediately after HDBR (R0). All groups are pooled together.

To explore relations between changes in $\dot{\text{V}}$ O_2_max and muscular parameters, we examined leg circumferences and lean mass. To explore relations between changes in $\dot{\text{V}}$ O_2_max and cardiovascular parameters, we looked at supine and upright SV, HR, CO, and TPR (Figure 10; Table 3).

**TABLE 3:** Correlations between percent changes in muscular and cardiovascular variables.

Correlation	Pearson r	*p* value
Muscular variables		
Thigh circumference—lean mass	0.64	0.002
Calf circumference—lean mass	0.69	0.0006
Cardiovascular variables		
$\dot{\text{V}}$ O_2_max—upright HR	−0.33	0.13
$\dot{\text{V}}$ O_2_max—upright SV	−0.30	0.18
$\dot{\text{V}}$ O_2_max—upright CO	−0.48	0.02
$\dot{\text{V}}$ O_2_max—upright TPR	0.12	0.61

Correlations between relative decreases in thigh and calf circumferences at D84 and relative decrease in lean mass at D85. Correlations between relative changes in $\dot{\text{V}}$ O_2_max at R + 2 and relative changes in upright heart rate, stroke volume, cardiac output, and total peripheral resistance measured during the tilt test immediately after HDBR (R0). All groups are pooled together.

Reductions in leg circumferences at D84 showed relatively strong positive correlations with a decrease in $\dot{\text{V}}$ O_2_max at R + 2 relative to Pre (Figures 10A,B). In addition, a decrease in leg circumferences strongly correlated with a decrease in lean mass (Table 3), supporting that leg circumference decreases occurred due to muscle loss (rather than fat loss). A decrease in supine SV, as well as an increase in supine HR, tended to correlate with a decrease in $\dot{\text{V}}$ O_2_max (Figures 10C,D) but to a lesser extent than that for leg circumferences. For upright SV and HR, we observed the absence of such correlations.

## 4 Discussion

For the first time, resistance exercise and running countermeasures were tested separately and compared directly during the same long-term HDBR. The main finding is that running countermeasure preserved $\dot{\text{V}}$ O_2_max, whereas resistance exercise limited its decrease. Both exercise protocols failed to limit cardiovascular deconditioning in terms of orthostatic intolerance, tachycardia, reduction in SV, and autonomic indices. Both countermeasures limited the loss in leg muscle volume (approximated by a decrease in leg circumferences), with more efficiency, again, for running. Both countermeasures limited the loss in lean mass but did not modify the increase in body fat. HDBR did not apparently affect circadian variance in profiles of BP, as 10% nocturnal BP dipping was preserved. Circadian BP decreased during HDBR (reduction of 6–7 mmHg for systolic BP), although without reaching statistical significance for “day” and “night” mean values. Subjects demonstrated only a modest decrease in orthostatic tolerance post-HDBR, including those in the control group.

Our discussion will mainly focus on comparison with the results of other long-term bedrest protocols (>1 month). Some of them have tested combined physical exercise countermeasures (Trappe et al., 2007; Schneider et al., 2009; Coupé et al., 2011; Lee et al., 2014; Ploutz-Snyder et al., 2018).

### 4.1 Effects of prolonged strict HDBR

#### 4.1.1 Maximal oxygen uptake

We aimed to explore $\dot{\text{V}}$ O_2_max dynamics specifically during HDBR, so we have chosen $\dot{\text{V}}$ O_2_max testing in the supine position. For recovery measurements, we have chosen to perform the orthostatic tolerance test on the first day out of bed, and $\dot{\text{V}}$ O_2_max testing—the second day out of bed. Aerobic capacity expectedly decreased during bedrest, presumably due to enhanced physical inactivity, with rapid initial loss at M1 (−21%), followed by slower reduction, reaching −26% at M3. Compared to other bedrest studies, the review by Lee et al. (2010) estimates the average $\dot{\text{V}}$ O_2_max decrease rate for the first month of bedrest to be 0.8%–0.9% per day (i.e., a 24%–27% decrease for 30 days), with progressive slowing afterward. Furthermore, the pre-to-post decrease in $\dot{\text{V}}$ O_2_max in the group without countermeasures (*n* = 9 M) at the end of the 90-day HDBR performed in 2001–2002 in Toulouse was 32 ± 7% (Belin de Chantemele et al., 2004; Capelli et al., 2006). Thus, the $\dot{\text{V}}$ O_2_max loss in our HDBR was slightly less than expected. This may be related to the lower initial fitness of our subjects (baseline $\dot{\text{V}}$ O_2_max of ∼32 ml/kg/min) than usually seen in bedrest protocols (baseline $\dot{\text{V}}$ O_2_max of 40 ml/kg/min and more) (Capelli et al., 2006; Ried-Larsen et al., 2017).

In long-term spaceflights, a maximal decrease in $\dot{\text{V}}$ O_2_max (approximately 17%) occurs during the first 2 weeks of the ISS mission (Moore et al., 2014) and then slightly improves during the 5–6 mo flight, remaining at a ∼15% decrease (Moore et al., 2014; Hoffmann et al., 2016). Compared to that during flight, the $\dot{\text{V}}$ O_2_max loss in our HDBR was larger. This may be related to the inflight mandatory daily exercise.

#### 4.1.2 Orthostatic tolerance during tilt test

In our study, 90 days of strict bedrest did not significantly reduce the tolerance time, although two subjects in the control group did not reach 10 min in the orthostatic position during the tilt post-HDBR (one of them was nonfinisher pre-HDBR). Interestingly, in a previous 60-day HDBR with Chinese subjects, tolerance was also preserved (Coupé et al., 2011). That 60-day HDBR was not strict—subjects were allowed to stand briefly in the morning and in the evening for hygiene. Our 90-day study was a strict HDBR, so a short period of orthostatic stimulation could not be evoked. As orthostatic tolerance is dependent on head-to-heart distance, shorter subjects might better tolerate orthostasis (Ludwig and Convertino, 1994; Pavy-Le-Traon et al., 1999). In line with this, Table 4 summarizes the characteristics of the subjects in different studies who participated in long-term bedrest, suggesting that shorter subjects are more tolerant of tilt.

**TABLE 4:** Height and orthostatic tolerance after long-term bedrest without countermeasures in men.

Bedrest duration (d)	Control subjects (M)	Height, cm mean ± SEM	Test type	Non-finishers	References
42	7	176 ± 1	6-min sit+10-min stand	4/7	Pavy-Le Traon et al. (1999)
90	9	173 ± 1	10-min 80° tilt	4/9	Belin de Chantemele et al. (2004)
60	7	169 ± 1	20-min 75° tilt	2/7	Coupé et al. (2011)
35	9	180 ± 1	0.5-min sit+12-min stand	4/9	Adami et al. (2013)
90	8	170 ± 1	30-min 75° tilt	2/8	Present study

#### 4.1.3 Body composition: Muscle atrophy and gain in fat

Body mass did not change significantly, but mass repartition shifted from lean to fat, together with a decrease in leg circumferences, presumably due to enhanced inactivity.

Considering leg muscle loss, previous HDBR studies using magnetic resonance imaging (MRI) found, in no-exercise groups, a volume loss for quadriceps of −18% (90-d HDBR in men) (Alkner and Tesch, 2004) and −21% (60-d HDBR in women) (Trappe et al., 2007); for triceps surae, both studies found a loss of −29%. In our study, as change in volume for the leg segment (roughly cylindrical) is proportional to the squared change in circumference, in the control group, estimated volume loss consisted of −14% for the thigh and −19% for the calf. Taken together, this suggests that calf muscles are more influenced by long-term HDBR than thigh ones. Other studies also noted a hierarchy of greater atrophy in the extensors of the calf, thigh, and then hip musculature (Belavý et al., 2009). Lesser values for our study might be explained by difference in methods (direct MRI measurement vs. approximation *via* circumference measurement), and also by difference in muscles taken into account (targeted measure of extensors which are more affected by unloading vs. the total cross-section).

#### 4.1.4 Cardiovascular response during tilt test

An observed increase in resting and upright HR post-HDBR is commonly reported after spaceflight and microgravity simulations as a part of cardiovascular deconditioning syndrome. The post-HDBR increase in resting BP before orthostatic testing, frequently seen in bedrest protocols, could be associated with the stress of the first orthostatic challenge after prolonged bedrest (Traon et al., 1998). Decrease in resting SV (of ∼30%) reflects bedrest-induced hypovolemia.

#### 4.1.5 Day–night heart rate and blood pressure

Day–night rhythms of HR and BP were visibly preserved. Of note, for technical reasons, we were limited to continuous recordings of 21 h. We did not perform specific circadian analysis. However, a recent study specifically evaluating circadian rhythms of R-R intervals in 5-, 21-, and 60-day HDBR using Cosinor analysis (Solbiati et al., 2021) revealed flattening of day–night oscillations for R-R intervals progressing with the duration of bedrest. These results are in agreement with our findings.

**FIGURE 11:**
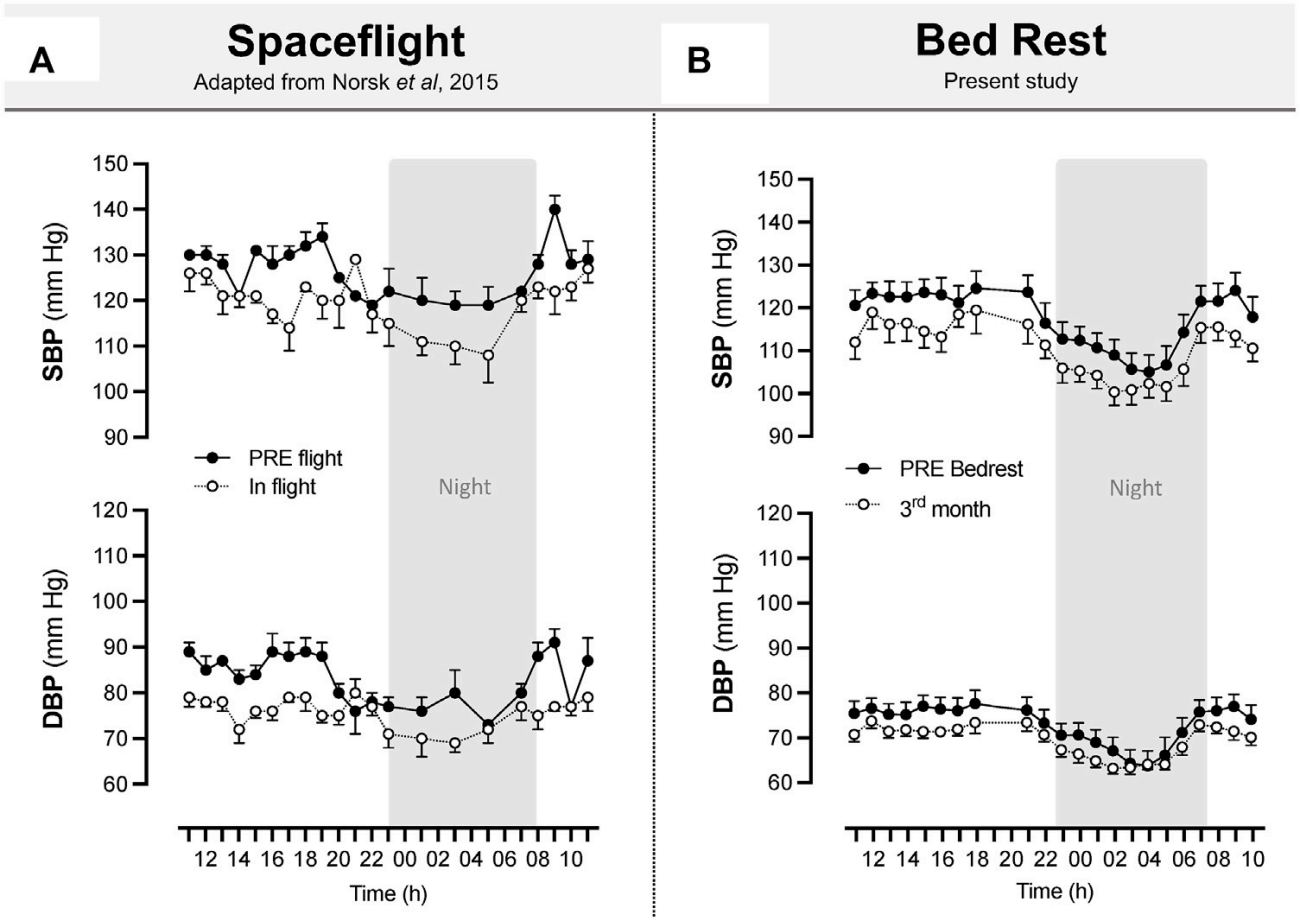
Space flight and long-term bedrest similarly induced a decrease in systolic and diastolic blood pressure. Systolic and diastolic blood pressure during spaceflight **(A)**. Upper arm artery blood pressure was measured between 85 and 192 days of flight. Recordings are hourly during the day and bihourly during the night over 11:00–11:00. The sample size was *n* = 8, and data are presented as the mean ± SEM. *Adapted from* *Norsk et al., 2015*. Systolic and diastolic blood pressure during 90-day bedrest **(B)** estimated from Pulse Transit Time using the SOMNOtouch™ device. Recordings are beat by beat over 21:00–18:00. The sample size was *n* = 8 (CON), and data are presented as the mean ± SEM from the present study.

Interestingly, circadian blood pressure in the present study exhibited the same decreasing pattern in the control group during HDBR (drop of 6–7 mmHg for systolic blood pressure) as that observed in long-duration spaceflight [8–10 mmHg reported by Norsk et al. (2015)] (Figure 11). The healthy night “dip” in systolic and diastolic blood pressure is preserved in bedrest, as was also observed by Norsk et al*.* (2015) in real microgravity. This reinforces our belief that long-term ground-based microgravity simulations are useful in the context of preparing for missions to the Moon and Mars.

### 4.2 Effects of physical exercise countermeasures

#### 4.2.1 Muscular system and body composition

In space, training is seen as a holistic method and used in an integrative way, with aerobic, mainly locomotor, modality to maintain the cardiorespiratory fitness and resistance modality focusing on strength and muscle size. With future goals of reaching the Moon and Mars, solutions to apply both aerobic and resistance exercise modalities inevitably present issues. These primarily relate to reducing the mass, size, energy use, heat production, and cost of devices (Steele et al., 2019).

Leg muscles are more strongly affected in microgravity, as astronauts still use their arms and hands to conduct daily routines and navigate across the ISS. In this study, RUN and RES countermeasures halved LM loss and limited lower limb circumferences loss. Consistently, after 60 days of bedrest in women, DEXA measurements revealed significantly greater leg lean mass in the running + resistance exercise group (supine treadmill within LBNP 3–4 days/wk and supine leg- and calf-press exercises 2–4 days/wk) than in no-exercise controls (12.5 vs. 11.6 kg) (Lee et al., 2014), and whole-body lean tissue mass loss was limited (∼5% loss in controls vs. ∼2% loss in exercise group) (Schneider et al., 2009).

#### 4.2.2 $\dot{V}$ O_2max_

Changes in the muscular $\dot{\text{V}}$ O_2_ kinetics, indicating aerobic detraining effects, can be present for up to 21 days following spaceflight (Hoffmann et al., 2016). If it persists on return to gravity, this condition may appear to be a source of concern for crews, so physical exercise countermeasures need to be evaluated. It has been shown that combined resistance (3 d/wk) and aerobic (6 d/wk) exercise can maintain peak aerobic capacity after 70 days of bedrest (Ploutz-Snyder et al., 2018). Furthermore, in 60-day bedrest, a high-intensity jump countermeasure (5–6 sessions/wk, 8–17 min/session), preserved resting HR and $\dot{\text{V}}$ O_2_peak (Kramer et al., 2017), the major criteria for cardiovascular deconditioning.

The present study is the first to directly compare running alone (without being coupled to nutritional, pharmacological, or other exercise countermeasures) and resistance exercise alone. We showed that the RUN succeeded in maintaining the baseline $\dot{\text{V}}$ O_2_max, while the resistance countermeasure limited $\dot{\text{V}}$ O_2_max loss.

#### 4.2.3 Orthostatic tolerance, muscle mass, indices of CV, and autonomic fitness

The RUN group had four intolerant subjects post-HDBR compared with 2 in CON and 2 in RES. Neither countermeasure counteracted orthostatic intolerance. Of note, exercise training might increase limb compliance and thus reduce orthostatic tolerance; on the other hand, if initial tolerance is low, moderate exercise might also improve it (Pavy-Le-Traon et al., 2007). In any case, the subjects of all 3 groups maintained rather good orthostatic tolerance after 90 days of strict bedrest.

Both countermeasure groups lost less global lean mass and leg circumferences than the control group, so these types of exercise could limit muscle wasting.

In our study, both resistance and running countermeasures had no effect on volemia (SV) and the hemodynamic and autonomic indices associated with cardiovascular fitness. Consistently, previous 90-day bedrest with the same resistance exercise device (flywheel) also failed to improve these cardiovascular parameters (Belin de Chantemele et al., 2004). In another study (60-day bedrest), resistance exercise combined with vibration prevented an increase in the sympathetic index and limited a drop in baroreflex sensitivity (Coupé et al., 2011), although it had no effect on orthostatic tolerance. Furthermore, aerobic exercise coupled with restoration of plasma volume maintained orthostatic tolerance after 18 days of bedrest (Shibata et al., 2010).

Exercise intensity in our protocol might appear insufficient in terms of time and/or power. Thus, on the ISS, astronauts perform 1 h of resistance and 30–45 min of locomotor aerobic exercises 6 days/wk (Wang et al., 2019). In addition, a combination of countermeasures is conceivable. According to Wang et al. (2019), artificial gravity is considered the first option for the novel concept of countermeasures for Chinese long-term spaceflight. In fact, artificial gravity protocols were able to limit orthostatic tolerance loss after short bedrest (Stenger et al., 2012; Li et al., 2017). However, they seemed less efficient in abolishing cardiovascular deconditioning (in terms of tachycardia, reduction in LVEDV, CO, and SV) after long-term HDBR (Hoffmann et al., 2021).

In the current bedrest, a novel combined resistance exercise platform has been tested. Another flywheel exercise device, developed in Sweden by Alkner and Tesch almost 20 years ago, has been tested in bedrest studies and appeared very efficient in conserving the knee extensor but not the knee flexor muscles during 90-day HDBR (LTBR study, France, 2001–2002, *n* = 9 in Flywheel exercise group and *n* = 16 in inactive group, Belavý et al., 2017).

Comparison between these two systems is clearly limited by the differences in protocols as well as by the absence of direct results on muscles in the current study. However, in terms of time spent on resistive exercise, LTBR subjects exercised for 20 min every third day, that is, 2-3 times/wk, which seems less than our RES subjects (for 45 min 3 times/wk). In terms of efficiency, reducing muscle atrophy seems comparable. Indeed, for calf muscles, LTBR resistive exercise reduced soleus loss by ∼31% (20% volume loss for flywheel group vs. 29% loss in inactive group), whereas in the current bedrest, RES exercise reduced calf volume loss by ∼26% (14% estimated loss in RES vs. 19%—in Controls). For thigh muscles, LTBR, resistive exercise reduced vasti loss by ∼80% (4% volume loss for flywheel group vs. 20% loss in inactives). In our bedrest, RES exercise reduced thigh volume loss by ∼71% (4% estimated loss in RES vs. 14%—in Controls).

It should be recognized that the effect of any training depends not only on its modality but also on the specific parameters of the exercises used (mode, intensity, and volume). For example, as shown in a 370-day 4.5° HDBR with 10 healthy men, intense treadmill training can overshadow the effect of weak resistance training (Grigoriev and Kozlovskaya 2018). So we can compare for the chosen modes of running and resistance training. But all specific parameters of exercise are extremely important, and this makes for very delicate comparison of exercise countermeasures in general.

### 4.3 Variables potentially related to maximal oxygen uptake decrease

The decrease in $\dot{\text{V}}$ O_2_max after this long-term HDBR appears to be mainly related to muscular parameters, with a positive correlation between the decrease in leg circumferences and decrease in $\dot{\text{V}}$ O_2_max. Moreover, the presence of a strong correlation between the dynamics of leg circumferences and lean mass suggests that the decrease in circumferences indeed reflects muscle loss. Correlations between the decrease in $\dot{\text{V}}$ O_2_max and other cardiovascular changes appear weaker. Decrease in $\dot{\text{V}}$ O_2_max tended to correlate with decrease in supine SV and with increase in supine HR. But we observed the absence of such correlations for upright SV and HR.

Thus, higher correlation coefficients observed with muscular parameters indicate a stronger dependency of $\dot{\text{V}}$ O_2_max decrease post-HDBR on impairment of muscular functions. Muscular parameters better explain the decrease in $\dot{\text{V}}$ O_2_max at later stages of HDBR and seem to be more closely related to this decrease.

During bedrest, the early $\dot{\text{V}}$ O_2_max decrease is mainly due to cardiovascular deconditioning (Capelli et al., 2006). In the present study, we did not perform hemodynamic tests before the end of the first month. However, Capelli and coworkers postulated that the later phase of $\dot{\text{V}}$ O_2_max decreases after long-term bedrest can be due to muscle function deterioration through the impairment of peripheral gas exchanges (Capelli et al., 2006). This hypothesis is in line with our findings.

### 4.4 Limitations

Several limitations should be noted in this study. The main concern is the lack of direct measures for muscle outcomes (using MRI, DEXA, etc.). Indeed, leg circumferences are not a strong measure of regional muscle mass, cross-sectional area, or architecture. The sample size was limited to eight subjects in the control group and seven in the exercise groups. This small *n* has an impact on statistical power. Additionally, plasma volume was not measured directly, so we used resting stroke volume changes as an indirect reflection of plasma volume changes, but this remains a simple estimation and not an actual measurement. Moreover, we did not perform cardiac structure tests (especially assessments of left ventricular mass evolution) and thus did not study the role of eventual cardiac muscle atrophy in $\dot{\text{V}}$ O_2_max decrease.

## 5 Conclusion

During this 90-day strict head-down bedrest, running exercise countermeasure preserved $\dot{\text{V}}$ O_2_max, whereas resistance exercise limited its decrease. Both exercise protocols failed to limit cardiovascular deconditioning in terms of orthostatic intolerance, tachycardia, reduction in SV, and autonomic indices. The decrease in $\dot{\text{V}}$ O_2_max after this long-term HDBR appears to be mainly related to muscular parameters, with a positive correlation between the decrease in leg circumferences and decrease in $\dot{\text{V}}$ O_2_max. Muscular training countermeasures should be maintained for the entire duration of deep space missions to counteract a progressive decline in overall capability to work.

## Data Availability

The raw data supporting the conclusion of this article will be made available by the authors, without undue reservation.

## Abbreviations

ACC, Astronaut Center of China; BP, blood pressure; CM, countermeasure; CO, cardiac output; CON, control group; DBP, diastolic blood pressure; ECF, extracellular fluid; ECG, electrocardiogram; FM, fat mass; HDBR, head-down bedrest; HF, high frequency; HR, heart rate; HRV, heart rate variability; ICF, intracellular fluid; ISS, International Space Station; LBNP, lower body negative pressure; LM, lean mass; LF, low frequency; OTT, orthostatic tolerance time; RES, resistance exercise group; RUN, running exercise group; SBP, systolic blood pressure; SEM, standard error of the mean; Sup, supine; SV, stroke volume; TBW, total body water; TPR, total peripheral resistance.
