# Supplementary material for: Running vs. resistance exercise to counteract deconditioning induced by 90-day head-down bedrest
Source: Front Physiol. 2022 Aug 31;13:902983. doi: 10.3389/fphys.2022.902983 (PMC9473647; doi:10.3389/fphys.2022.902983)
Supplement: Supplementary file 1 [file Table1.pdf]

Supplementary Data 1. Evolution in maximal oxygen uptake ($\dot{V}O_2max$) during HDBR.

	Group	D43	D71	R+2	global **ANOVA**
**$\dot{V}O_2max$,** ΔmL.kg^{-1}.min^{-1}	CON	-7.0±1.2 #	-8.3±0.8 #	-8.5±0.9 #	*Bedrest p<0.0001* *CM p=0.004*
	RES	-2.6±0.8* #	-3.6±1.2*	-5.1±1.1 #	
	RUN	-0.6±1.2*	-2.2±0.9*	-1.6±1.3*	
**$\dot{V}O_2max$,** ΔL.min^{-1}	CON	-0.5±0.1 #	-0.6±0.1 #	-0.6±0.1 #	*Bedrest p<0.0001* *CM p=0.0002*
	RES	-0.2±0.0* #	-0.2±0.1* #	-0.3±0.1* #	
	RUN	-0.1±0.1*	-0.2±0.1*	-0.2±0.1*	

Variations (mean±SEM) are calculated as Δ=value $_{post}$ – value $_{pre}$
*p<0.05 vs. CON; #p<0.05 vs. Pre

Supplementary Data 2. Evolution in body mass, body composition and fluid compartments during HDBR.

	Group	D30	D57	D85	global **ANOVA**
**Body mass,** Δkg	CON	-0.8±0.4	-1.2±0.9	-2.0±1.0	*Bedrest p=0.39* *CM p=0.19*
	RES	0.7±0.6	0.7±0.7	0.4±0.9	
	RUN	-0.9±0.5	-0.4±0.8	-0.4±0.9	
**Fat mass,** Δkg	CON	2.5±0.5 #	3.5±0.4 #	4.0±0.7 #	*Bedrest p<0.0001* *CM p=0.34*
	RES	1.3±0.4	2.5±0.5 #	3.2±0.5 #	
	RUN	1.4±0.6	2.3±0.7	3.7±0.8 #	
**Lean mass,** Δkg	CON	-3.3±0.6 #	-4.7±0.8 #	-5.9±1.1 #	*Bedrest p<0.0001* *CM p=0.008*
	RES	-0.6±0.3*	-1.7±0.5* #	-2.7±0.6 #	
	RUN	-1.6±0.7	-2.0±0.4* #	-3.3±0.7 #	
**Total body water,** ΔL	CON	-2.4±0.4 #	-3.5±0.6 #	-4.3±0.8 #	*Bedrest p<0.0001* *CM p=0.008*
	RES	-0.4±0.2*	-1.3±0.4* #	-2.0±0.4 #	
	RUN	-1.2±0.5	-1.4±0.3* #	-2.4±0.5 #	
**Extracellular fluid,** ΔL	CON	-0.9±0.2 #	-1.0±0.2 #	-1.0±0.2 #	*Bedrest p<0.0001* *CM p=0.37*
	RES	-0.3±0.2	-0.6±0.4	-0.6±0.2	
	RUN	-0.7±0.2	-0.9±0.4	-0.8±0.2	
**Intracellular fluid,** ΔL	CON	-1.6±0.3 #	-2.5±0.4 #	-3.3±0.6 #	*Bedrest p<0.0001* *CM p=0.001*
	RES	-0.1±0.1*	-0.7±0.3*	-1.4±0.2* #	
	RUN	-0.5±0.3*	-0.5±0.2*	-1.7±0.3 #	

Variations (mean±SEM) are calculated as Δ=value $_{post}$ – value $_{pre}$
*p<0.05 vs. CON; #p<0.05 vs. Pre

Supplementary Data 3. Evolution in thigh and calf circumferences during HDBR.

	**Group**	**D28**	**D56**	**D84**	global **ANOVA**
**Thigh circumference,** Δcm	CON	-2.2±0.5 #	-3.0±0.4 #	-3.5±0.5 #	*Bedrest p<0.0001*<br>*CM p=0.0003*
	RES	-0.9±0.2 #	-0.8±0.3*	-1.0±0.4*	
	RUN	-0.2±0.4*	-0.4±0.4*	-0.3±0.5*	
**Calf circumference,** Δcm	CON	-1.7±0.2 #	-3.2±0.2 #	-3.8±0.2 #	*Bedrest p<0.0001*<br>*CM p=0.0001*
	RES	-1.4±0.3 #	-2.3±0.2* #	-2.5±0.3* #	
	RUN	-0.8±0.1* #	-1.5±0.2* #	-1.8±0.2* #	

Variations (mean±SEM) are calculated as $\Delta = \text{value}_{post} - \text{value}_{pre}$
*p<0.05 vs. CON
